# Postmortem evidence of cerebral inflammation in schizophrenia: a systematic review

**DOI:** 10.1038/mp.2016.90

**Published:** 2016-06-07

**Authors:** M O Trépanier, K E Hopperton, R Mizrahi, N Mechawar, R P Bazinet

**Affiliations:** 1 Department of Nutritional Sciences, Faculty of Medicine, University of Toronto, Toronto, ON, Canada; 2 Research Imaging Centre, Centre for Addiction and Mental Health, Toronto, ON, Canada; 3 Institute of Medical Science, University of Toronto, Toronto, ON, Canada; 4 Department of Psychiatry, University of Toronto, Toronto, ON, Canada; 5 McGill Group for Suicide Studies, Douglas Mental Health University Institute, Montreal, QC, Canada; 6 Department of Psychiatry, McGill University, Montreal, QC, Canada

## Abstract

Schizophrenia is a psychiatric disorder which has a lifetime prevalence of ~1%. Multiple candidate mechanisms have been proposed in the pathogenesis of schizophrenia. One such mechanism is the involvement of neuroinflammation. Clinical studies, including neuroimaging, peripheral biomarkers and randomized control trials, have suggested the presence of neuroinflammation in schizophrenia. Many studies have also measured markers of neuroinflammation in postmortem brain samples from schizophrenia patients. The objective of this study was to conduct a systematic search of the literature on neuroinflammation in postmortem brains of schizophrenia patients indexed in MEDLINE, Embase and PsycINFO. Databases were searched up until 20th March 2016 for articles published on postmortem brains in schizophrenia evaluating microglia, astrocytes, glia, cytokines, the arachidonic cascade, substance P and other markers of neuroinflammation. Two independent reviewers extracted the data. Out of 5385 articles yielded by the search, 119 articles were identified that measured neuroinflammatory markers in schizophrenic postmortem brains. Glial fibrillary acidic protein expression was elevated, lower or unchanged in 6, 6 and 21 studies, respectively, and similar results were obtained for glial cell densities. On the other hand, microglial markers were increased, lower or unchanged in schizophrenia in 11, 3 and 8 studies, respectively. Results were variable across all other markers, but SERPINA3 and IFITM were consistently increased in 4 and 5 studies, respectively. Despite the variability, some studies evaluating neuroinflammation in postmortem brains in schizophrenia suggest an increase in microglial activity and other markers such as SERPINA3 and IFITM. Variability across studies is partially explained by multiple factors including brain region evaluated, source of the brain, diagnosis, age at time of death, age of onset and the presence of suicide victims in the cohort.

## Introduction

Schizophrenia is a psychiatric disorder which affects ~0.5 to 1% of the population in their lifetime.^1, 2^ Psychosis normally arises in the late teenage years or early adulthood, between 18 and 25 years of age.^3^ Although the cause underlying this mental illness remains to be elucidated, several biological factors have been proposed, including abnormalities in oligodendrocytes,^4, 5^ N-methyl-D-aspartate (NMDA) signaling^6^ and dopaminergic transmission.^7^

Neuroinflammation has been suggested to be a potential contributor in the pathogenesis of the schizophrenia.^8, 9, 10, 11^ Classically, the brain is considered to be immunologically privileged due to the blood–brain barrier limiting cell entry.^12^ Under normal conditions, microglia, the resident immune cells of the brain, are found in a ramified (‘resting') state, surveying the environment. Following injury or the exposure to pro-inflammatory signals such as interferon (IFN)-γ and tumor necrosis factor (TNF)-α, ramified microglia can become activated and release pro-inflammatory cytokines such as interleukin (IL)-1β, IL-6, IFN-γ or chemokine (c-x-c motif) ligand (CCL) 11.^13^ Microglia also increase the expression of cyclooxygenase (COX)-2, an enzyme involved in the arachidonic cascade, which can lead to the production of the pro-inflammatory lipid mediator prostaglandin E_2._^14^ Pro-inflammatory cytokines released from microglia, such as IL-1β, can activate astrocytes. In turn, activated astrocytes also have the ability to release pro-inflammatory cytokines and chemokines, such as IL-1β, CCL5 and TNF-α,^15^ and typically display increased glial fibrillary acidic protein (GFAP) expression.^16^

Evidence has accumulated supporting a link between inflammation and schizophrenia. Serum or plasma concentrations of pro-inflammatory markers have been investigated in several studies. Two meta-analyses illustrate that IL-6 is consistently elevated in serum and plasma of patients with schizophrenia,^17, 18^ whereas IL-1β and TNF-α were found to be increased in one meta-analysis,^18^ but not in the other.^17^ Genetic studies have also linked polymorphisms in major histocompatibility complex (MHC) regions with risk of schizophrenia.^19, 20^

Neuroinflammation has also been associated with schizophrenia. Advancements in *in vivo* PET imaging has enabled imaging of neuroinflammation in schizophrenic patients.^21^ However, studies imaging the translocator protein 18 kDA (TSPO), a marker of activated microglia, have yielded mixed results. Early studies utilizing the TSPO ligand [^11^C]PK11195 suggested that schizophrenic patients have higher levels of activated microglia compared with healthy controls.^22, 23^ More recent studies, using second-generation TSPO ligands, however, had mixed results, with some reporting increased microglia activation in schizophrenia,^24^ whereas others failed to replicate earlier studies and found no difference between patients and healthy controls.^25, 26^ The reasons for the disparities between studies are not clear, but likely related to different TSPO ligands used or different samples studied across research groups.

As schizophrenia has been associated with inflammation, attempts have been made to treat symptoms with non-steroidal anti-inflammatory drugs (NSAID) as an add-on therapy to conventional treatments. Although some studies found added benefits of NSAID on symptoms,^27, 28, 29^ one study did not show any beneficial effects.^30^ A meta-analysis of five published and three non-published studies found no effect of NSAID on the Positive and Negative Syndrome Scale total scores, but did detect a small yet statistically significant beneficial effect of NSAID add-on therapy for the treatment of positive symptoms.^31^ Omega-3 polyunsaturated fatty acids (n-3 PUFA), which are also thought to have anti-neuroinflammatory properties,^32, 33^ have also yielded mixed results in the treatment of schizophrenia. Administration of 3 g per day of n-3 PUFA in combination with 300 mg per day of alpha-lipoic acid for up to 2 years did not decrease the relapse rate of schizophrenic patients.^34^ An earlier report, however, found that administration of n-3 PUFA was beneficial in reducing the conversion of subthreshold psychosis to a first episode psychotic event in adolescents.^35^

It is unclear whether neuroinflammation associated with schizophrenia is causing or is a result of the disorder. It has been suggested that microglia activation and cytokine release could lead to neuronal and glial injury,^36^ resulting in dopaminergic and glutaminergic system dysregulation.^37, 38^ Neurogenesis and synapse connectivity may also be affected by neuroinflammation.^39, 40^ Moreover, activation of astrocytes may also cause abnormal production of kynurenic acid and upregulate the expression of glutamate transporters.^9, 11, 41^

Despite the mixed results in both *in vivo* imaging and clinical trials, it appears plausible that inflammation may have a role in schizophrenia. Numerous postmortem studies have measured pro-inflammatory markers in patients suffering from schizophrenia. To date, no systematic review of the field has been published on the topic. This article set out to systematically characterize the literature on neuroinflammation as measured in postmortem brains from schizophrenia patients.

## Materials and methods

We performed a systematic search for literature indexed in MEDLINE, Embase and PsycINFO up to 20th March 2016. Full search criteria can be found in the Supplementary Materials. Only peer-reviewed primary research articles were considered as eligible studies. References of yielded articles were searched for possible eligible articles that were missed by the search.

Once duplicate articles were removed, studies were screened based on title and abstract for several components including studies which were on schizophrenia and (1) carried out with postmortem brain samples, (2) measured neuroinflammatory markers and (3) were compared with matched psychiatrically and neurologically healthy controls. Studies evaluating markers of astroglia, microglia, gliosis, cytokines, arachidonic acid cascade and substance P were included (for full search terms, see Supplementary Materials). Other markers were considered if the authors referred to their implication in neuroinflammation. Although not always stated by the authors as a microglial marker, MHC (also know as human leukocyte antigen, HLA) complex I and II were both considered as possible microglial markers as both have been shown to be elevated in microglia.^42^ Untargeted approaches, such as microarray and shotgun proteomics, were also excluded unless targeted approaches were used to confirm the results. Viruses and infection were not considered for this review and were excluded. Reviews were searched for relevant articles, but themselves were excluded from the results. Finally, non-English papers and conference abstracts were also excluded.

Articles were evaluated and data were extracted onto an electronic data extraction form by MOT. Extractions were confirmed by a second independent reviewer (KEH). From eligible studies, number of subjects, sex, race, duration of illness, onset of illness, postmortem interval, freezer time, death from suicide, substance abuse, medication, RNA quality and brain pH were extracted as background information. Unless specifically stated, suicide was not assumed as cause of death. Study design information, such as neuroinflammatory markers measured, measuring techniques and in which brain regions the measurements were made were all extracted, along with comparative results between schizophrenia and healthy controls. Thus, all results discussed below are relative to controls unless otherwise stated.

## Results

**Figure 1:**
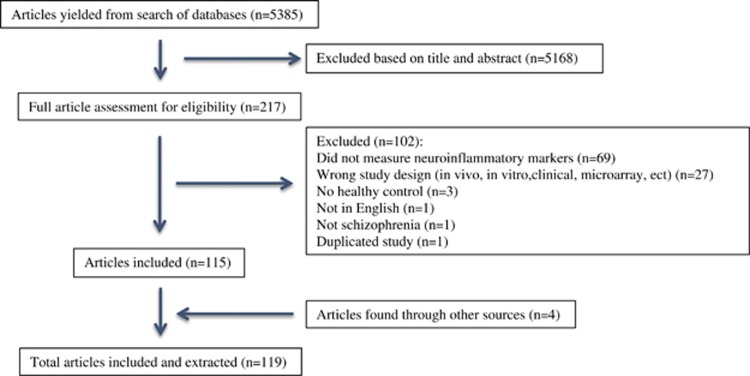
Systematic search results.

Following removal of duplicates, the search yielded 5385 unique results. A total of 5168 articles were excluded based on either title or abstract. The remaining 217 articles were fully screened for potential inclusion. Out of those remaining 217 articles, only 115 articles met the inclusion criteria. Four more articles were found in the reference section of papers yielded from the search (Figure 1).

## Astroglia

Our search yielded a total of 42 studies which assessed astrocytes in postmortem brain in schizophrenia (Table 1).

Of those 42 studies, 33 studies evaluated potential differences in astrocytes in schizophrenia by measuring GFAP expression or immunoreactive distribution. Out of the 33 studies evaluating GFAP expression, 21 did not detect any schizophrenia-associated changes, 6 studies reported a decrease in GFAP expression, whereas 6 studies reported increased expression.

The first study to evaluate GFAP was published in 1986 by Robert *et al.*^43^ In their study of the temporal cortex of 5 schizophrenic patients, immunohistochemical analysis found no differences in GFAP staining in schizophrenia brains compared with healthy controls,^43^ and was confirmed in a subsequent study with a larger cohort.^44^ Similarly, many quantitative immunohistochemical studies found no differences in GFAP cell density in several other brain regions including the hippocampus,^45, 46^ amygdala,^47, 48^ subiculum,^45, 49^ mediodorsal thalamus,^50^ caudate,^50, 51^ periventricular nucleus,^51^ nucleus basalis,^52^ premotor cortex,^49^ dorsolateral prefrontal cortex,^53^ midfrontal cortex,^45, 46^ orbitofrontal cortex,^45, 46^ entorhinal cortex,^45, 46, 47, 49, 54^ visual cortex,^45^ calcarine cortex^46^ and anterior cingulate cortex.^53^ When compared with Alzheimer's and Huntington's disease patients, schizophrenic patients had lower GFAP-labeled cells.^43, 45, 46^ However, schizophrenic patients presenting with dementia had significantly higher GFAP cell density than schizophrenic patients without dementia in multiple brain regions including hippocampus, entorhinal cortex and orbitofrontal cortex.^45^ GFAP was also reported to be correlated with age.^54^ Although Hercher *et al.*^55^ also found no differences in GFAP cell density in the dorsolateral prefrontal cortex in schizophrenia, they did find a decrease in GFAP fraction area and increased clustering. Phosphorylated GFAP was investigated in one immunohistochemical study. In a cohort of 15 patients, no difference in phosphorylated GFAP was observed between schizophrenic brains and those of healthy controls in the hippocampus.^56^ The authors did note, however, a decrease in phosphorylated GFAP-labeled cells in the dorsolateral prefrontal cortex next to blood vessels.^56^

Similar to the immunohistochemical studies mentioned above, multiple studies reported no increases in GFAP expression measured by other methods. No increase in GFAP mRNA expression was detected in the prefrontal^57^ and cingulate cortices^58^ of schizophrenics. Beasley *et al.*^59^ found no differences in GFAP in the anterior limb of internal capsule of schizophrenia compared with healthy controls as measured by enzyme-linked immunosorbent assay. Western blot analysis, similarly, found no increase in GFAP protein concentration in the cerebellum,^60, 61^ frontal cortex,^61, 62^ prefrontal cortex,^63, 64, 65^ visual cortex,^64^ occipital cortex,^61^ temporal cortex,^61^ parietal cortex,^62^ thalamus^61^ and pons^61^ of schizophrenic patients. Another study evaluating GFAP protein expression by western blot of various brain regions of 23 schizophrenics, including the dorsolateral prefrontal cortex, visual cortex, anterior cingulate cortex, hippocampus and temporal gyrus, failed to detect any changes in GFAP protein expression in schizophrenia, except for a significant decrease in the anterior cingulate cortex.^66^

A few studies have detected differences in GFAP protein expression. Williams *et al.*^67^ reported a decrease in GFAP cell density in the subgenual cingulate cortex and the corpus callosum in both the gray and white matter in a cohort of 10 schizophrenic patients compared to healthy controls. More specifically, another study found a decrease in number of fibrillary astrocytes in the subgenual anterior cingulate cortex.^68^ The authors, however, found no differences in gemistocytic astrocytes.^68^ In a separate study, the same group also found a decrease in GFAP cell density in the substantia nigra.^69^ Falkai *et al.*^49^ reported a decrease in GFAP cell density in the left inferior horn in men, whereas no effect was observed in women. On the other hand, Rajkowska *et al.*^70^ reported, in a cohort of 9 schizophrenic brains, an increase in GFAP cell density in layer V of the dorsolateral prefrontal cortex, whereas GFAP labeling area was reduced by 32%. These changes were layer specific, as no differences were detected in layer III and IV.^70^ This is slightly different from what Toro *et al.*^71^ observed, where an increase in GFAP, as measured by autoradiography, were observed in layers II, III and IV of the prefrontal cortex in schizophrenia. Importantly, this increase in GFAP was correlated with antipsychotic use. A decrease in GFAP in the orbitofrontal cortex was also observed.^71^ The authors proposed that the increase in prefrontal cortex was due to medication use whereas the decrease in the orbitofrontal cortex was due to the disease.^71^ Markova *et al.*^72^ reported increased GFAP positive cell area and reduced anisotropy, indicating gliosis, in the olfactory tubercle in schizophrenia. This is in agreement with another study where GFAP-labeled cells had changed in morphology in the prefrontal cortex of schizophrenics, being more stained and stunted, whereas also having a 2.4-fold increase in protein concentration and 30% increase in mRNA expression.^73^ Other studies have also shown that GFAP mRNA expression changes in schizophrenia. Barley *et al.*^74^ found that schizophrenic patients had increased GFAP mRNA expression in the putamen and mediodorsal thalamic nuclei. Like Toro *et al.*, increases in GFAP expression were correlated with duration of neuroleptic treatment.^74^ Although Catts *et al.*^75^ found no changes in GFAP mRNA expression in the dorsolateral prefrontal cortex between schizophrenic patients and healthy controls, a difference was observed in schizophrenia patients when they were stratified based on the presence of other neuroinflammatory markers including serpin peptidase inhibitor (SERPIN) A3, IL-1β, IL-6 and IL-8. Individuals with elevated neuroinflammation had a larger proportion of hypertrophic astrocytes compared with low neuroinflammation subjects.^75^ On the other hand, GFAP mRNA, as measured by riboprobe was decreased in the white matter of the anterior cingulate cortex.^76^ This effect, however, was not seen in the gray matter.^76^

Other astrocytic markers have also been measured in postmortem brain specimen of patients with schizophrenia. Hwang *et al.*^77^ showed increases in apolipoprotein 1 and adenosine A2A receptor mRNA expression, markers of perivascular astrocytes and implicated in inflammatory responses, in the hippocampus in schizophrenia. Similarly, along with increases in GFAP, schizophrenia was associated with increases in aldehyde dehydrogenase (ALDH)1 mRNA in several brain regions including the putamen, anteroventral nucleus, internal capsule and mediodorsal thalamic nucleus.^74^ In contrast, two other studies found no association between schizophrenia and ALDH1L1 mRNA measured in the deep layer of the cingulate cortex^58^ and protein concentration in the dorsolateral prefrontal cortex.^65^ Similar results were observed for GFAP and other astrocytic markers including vimentin,^58, 65^ excitatory amino-acid transporter (EAAT)1^65^ and phosphate-activated glutaminase.^58^ Katsel *et al.*,^58^ however, did find several other astrocytic markers, including S100b and EAAT2 mRNA to be downregulated in the cingulate cortex in schizophrenia. Differences in expression of various astrocytic markers may point to different types of astrocytes being affected in schizophrenia.^58^ S100b has been measured in a few other studies with mixed results. While one study found decreases in S100b protein measured by western blot analysis in the corpus callosum,^78^ another found no effect in several brain regions including Brodmann area (BA) 9, 10, 40 and 46.^62^ When separating paranoid schizophrenia from residual schizophrenia, one study found an increase in S100b-positive cells in paranoid schizophrenia compared with both residual schizophrenia and healthy controls in the dorsolateral prefrontal cortex.^79^ No effect was seen, however, in the white matter, as well as other brain regions such as hippocampus, mediodorsal thalamus, anterior cingulate cortex, superior temporal cortex and orbitofrontal cortex.^79^

Astrocytes have also been identified in postmortem brains by microscopic analysis with other staining techniques. Casanova *et al.*^80^ found no differences in astrocytes identified using Holzer's technique between the hippocampus of six schizophrenia patients and seven healthy controls. Similar to other studies comparing schizophrenic brains to those with Alzheimer's disease,^45, 46^ Alzheimer's disease brains had more astrocytes compared to both the schizophrenia and control groups.^80^ Similarly, stereological counting of Nissl stained astrocytes showed no differences in cell counts in the hippocampus,^81^ basolateral nucleus of the amygdala^82^ and pallidum.^82^ However, a significant decrease in astrocytes was measured in both the nucleus accumbens and mediodorsal thalamic nucleus.^82^

Changes in astrocytes in schizophrenia have also been investigated by electron microscopy.^83^ In a cohort of 19 schizophrenia patients, astrocyte morphology was unchanged in the hippocampus compared with healthy controls.^83^ However, when patients were separated based on age, increased astrocytes were observed in patients younger than 50 years old, but this effect was lost in older patients. On the other hand, astrocytic end feet were increased in both paranoid and non-paranoid schizophrenia in the prefrontal cortex,^84^ however, this effect was not present in the visual cortex in non-paranoid schizophrenics.^84^

## Microglia

From our search, a total of 22 articles reported on microglial markers in postmortem schizophrenic brains (Table 2). Out of these 22 studies, 11 studies reported an increase in microglial markers in postmortem brains, whereas 8 studies found no effect and 3 studies found a decrease in microglial markers.

Bayer *et al.*^85^ found that 3 of 14 schizophrenic patients had positive HLA-antigen D-related (DR) staining, MHC class II molecules involved in antigen presentation, whereas control subjects showed no staining in the hippocampus and frontal cortex. This is in agreement with two subsequent studies, where HLA-DR was increased in the prefrontal cortex,^86^ dorsolateral prefrontal cortex,^53^ superior temporal gyrus,^53^ inferior temporal gyrus^87^ and frontal lobe in schizophrenia.^87^ No changes, however, were seen in the cingulate cortex.^53^ This increase in HLA-DR labeling in the hippocampus appears to be more pronounced in paranoid schizophrenics, as this group has increased HLA-DR compared with both control and residual schizophrenics, although only significantly different from residual schizophrenics.^88^ Immunohistochemistry revealed differences in morphology of HLA-DR-labeled cells in schizophrenia, presenting a stunted and stronger labeling phenotype in the frontal cortex.^73^ It also has been reported that although patients show stronger HLA-DR labeling in the anterior cingulate cortex, microglia appear to be degenerating.^89^ Calprotectin, a member of the S100 family, co-expressed with microglial marker CD68 and was increased twofold in the dorsolateral prefrontal cortex in schizophrenic patients compared with healthy controls.^90^

Not all studies found significant differences in microglia density. Steiner *et al.*^91^ found no differences in HLA-DR protein in various brain regions between schizophrenia and healthy controls, but did note that the two individuals who committed suicide in their cohort did show more HLA-DR labeling. A follow-up study by the same group found a similar lack of effect of diagnosis, but that suicide was accompanied with higher HLA-DR-positive cells.^92^ In a microarray analysis, an increase in HLA-A, MHC I molecules, mRNA expression in the frontal cortex and superior frontal gyrus was observed between schizophrenia and healthy controls in the frontal cortex.^93^ This effect, however, was not statistically significant when mRNA expression was confirmed by qPCR.^93^ Schmitt *et al.*^94^ observed, in a microarray analysis of the temporal cortex of 10 schizophrenic patients and controls, lower mRNA expression of HLA-DRB3 and HLA-DPA1, subunits of HLA-DR, in schizophrenia. Similar to Saetre and colleagues, however, this effect was once again lost when analyzed by qPCR.^94^ Similarly, MHC II-positive cells were also unchanged in the subventricular zone in schizophrenia compared to healthy controls.^95^ Nakatani *et al.*^96^ also found no differences in HLA-DRA mRNA expression in the dorsolateral prefrontal cortex in schizophrenia, despite seeing a difference between control and bipolar disorder. Other microglial markers are also unchanged in schizophrenia. For example, ionized calcium-binding adapter molecule (Iba)1 as measured by immunohistochemistry showed no differences in microglial density in the cingulate cortex or dorsolateral prefrontal cortex.^55, 97^ Two prospective studies following patients who developed schizophrenia found no change in CD68 protein in the caudate nucleus,^50^ mediodorsal nucleus of the thalamus,^50^ hippocampus,^46^ and entorhinal^46^ and calcarine^46^ cortices in schizophrenic patients.

Similar decreases in HLA-DRA and HLA-DRB4 mRNA expression were observed in the temporal lobe.^98^ Despite not seeing changes in HLA-DR-positive cells, a separate study found microglial production of quinolinic acid was reduced in the hippocampus, and more specifically in the cornu ammonis (CA)1, of schizophrenic patients.^99^ MHC I protein concentration was lower in the dorsolateral prefrontal cortex in a non-smoking schizophrenic population, whereas no differences were seen in the orbitofrontal cortex.^100^ This effect was not seen in a smoking population.^100^ Systemic inflammation, however, appears to have a role in potential differences between patients with schizophrenia and healthy controls. In one study, schizophrenic patients with no systemic inflammation showed no differences as compared with healthy controls, but schizophrenics displaying systemic inflammation had lower HLA-A mRNA expression compared with psychiatrically healthy controls with systemic inflammation.^101^ However, when that same cohort was divided into smokers and non-smokers, regardless of systemic inflammation, HLA-B mRNA expression was increased in schizophrenic patients.^101^ The authors did report that HLA-A appeared to co-localize with glutaminergic neurons.^101^

## Undifferentiated glial cells

Multiple studies have evaluated glial cells in schizophrenia without the use of cell type-specific markers. Some studies separated the types of glial cells (that is, astrocytes, oligodendrocytes, microglia), as discussed previously. However, many studies using Nissl staining, evaluated the effect of schizophrenia on glial cells without differentiating between cell types. In total, 34 studies evaluated glial cells in schizophrenia, where 25 studies reported no difference, 7 studies found a decrease and 2 found an increase in glial cell densities (Table 3).

Stevens^102^ published the first study which met our inclusion criteria on the effect of schizophrenia on glial cells. In a cohort of 18 schizophrenic patients, fibrous gliosis measured by Holzer's staining was more pronounced in several brain regions including the hippocampus, hypothalamus, amygdala, thalamus, and periventricular areas compared to control. Comparable effects were observed in another study, which found increased fibrous gliosis as measured by Holzer's technique in the cerebral cortex of patients with schizophrenia.^103^

The increase in gliosis measured by Holzer's technique appears to differ, however, with a study published shortly after the report by Stevens and colleagues, which found, using a Nissl staining technique, a decrease in glial cell density in the CA3 and CA4 of the hippocampus.^104^ No effect of schizophrenia, however, was observed in the CA1 and subiculum.^104^ Similar decreases in glia were observed by Giemsa staining in the anterior cingulate cortex^105^ and by cresyl violet staining in the temporal cortex^106^ and planum temporale.^107^ A layer specific decrease in glial cell density measured by cresyl violet staining was observed in three studies, where effects were only in layer V of the dorsolateral prefrontal cortex,^108^ layer VI of the anterior cingulate cortex (statistical significance was lost following multiple corrections)^109^ and layer III of the motor cortex.^110^ The latter study did not detect any differences in both the prefrontal and cingulate cortices.^110^ Gliosis measured by [^3^H]PK11195 binding, a ligand which binds to the TSPO receptor found on activated microglia and astrocytes, was reduced in schizophrenia in the occipital cortex, parietal cortex, and putamen but not in the prefrontal cortex, temporal cortex, thalamus, pallidum, substantia nigra and caudate.^111^

Twenty-five studies, however, found no effect of schizophrenia on glial cell density in postmortem brains. In a study of 13 schizophrenic postmortem brains from the Stanley Foundation Neuropathology Consortium, Nissl staining revealed no differences in glial cell density or size in the amygdala.^112^ Similarly, no changes in glial density were obtained in the prefrontal,^113, 114, 115, 116^ frontal,^114^ subgenual prefrontal,^117^ occipital^113, 115^ and entorhinal^118^ cortices in schizophrenia compared with healthy controls. By comparison, Huntington's Disease had an ~50% increase in glial cell density compared with healthy controls.^113^ Moreover, Huntington's disease had increased density of larger glial cells.^115^ When glial cell density was measured by cresyl violet staining, no changes were detected between schizophrenia patients and healthy controls in several brain regions including the fusiform cortex,^119^ prefrontal gyrus,^120^ mediodorsal thalamic nucleus,^121^ layer III and V of the Heschl's gyrus,^122^ anterior cingulate cortex,^123, 124, 125^ prefrontal cortex,^125^ insular cortex,^126^ orbitofrontal cortex,^127^ hippocampus,^128^ planum temporale,^129^ substantia nigra^130^ and lateral geniculate nucleus.^131^ It should be noted that although Bogerts *et al.*^130^ failed to detect a difference in glial cell density in schizophrenia, they did report a significant reduction in glial size in schizophrenia patients. Gallocyanin, another staining technique, also did not detect an effect of schizophrenia on glial cell density in the dorsolateral prefrontal cortex of 13 male schizophrenic patients.^132^ Similarly, Beckmann and Lauer^133^ did not find any significant differences in glial density in several brain regions including the striatum, caudate, putamen and nucleus accumbens. Crow *et al.*^134^ also did not detect a differences in gliosis in the temporal horn and in the periventricular region using Holzer's technique between schizophrenia patients and controls. This was confirmed using diazepam inhibitor binding to evaluate gliosis. In another study, Nasrallah *et al.*^135^ found no differences in glial cell density in the corpus callosum in schizophrenia compared with healthy controls using hematoxylin and eosin staining. The authors did note that gliosis rating scores were higher in late onset schizophrenia compared with early onset and control patients.^135^

## Cytokines and chemokines

Ten studies evaluated cytokine and chemokine expression in postmortem brains of schizophrenic patients (Table 4). Two studies reported no difference in IL-1β mRNA in the prefrontal cortex,^86, 136^ despite measuring increases IL-1RA,^136^ IL-6 (ref. 86) and IL-8 mRNA.^86^ IFN-γ, measured by enzyme-linked immunosorbent assay, was reported to be increased in the prefrontal cortex of 35 schizophrenia patients compared to unaffected controls.^137^ However, Rao *et al.* reported 150% and 3.9 fold increases in IL-1β protein and mRNA respectively in the frontal cortex of schizophrenics. TNF-α protein and mRNA concentrations were also increased, 76% and 2.3-fold respectively, in schizophrenic patients.^73^ In a study of 19 schizophrenics, TNF-α receptor 1 mRNA was increased in the dorsolateral prefrontal and cingulate cortices compared to controls, whereas soluble TNF-α protein, transmembrane TNF-α protein and TNF-α receptor 2 mRNA concentrations were unchanged.^138^

A microarray analysis, followed by qPCR validation, found a decrease in IL-8 and IL-1α mRNA expression in the temporal cortex of 10 schizophrenic patients as compared with healthy control patients. However, increases detected in the microarray were not reproduced by qPCR for cytokines and chemokines such IL-1β, and CCL2.^94^ Another study also found a decrease in IL-8 mRNA in the middle frontal gyrus in schizophrenia, whereas IL-1β, TNF-α, IL-18 and IL-6 were not changed.^139^ Two more microarray studies also found decreases in expression, with CCL3 being reduced ninefold in the prefrontal cortex^96^ and IL-13RA reduced in the temporal lobe.^98^

## Arachidonic acid cascade

Seven studies have evaluated the arachidonic acid cascade in postmortem schizophrenic brains (Table 5).

Regional differences in concentration of cytosolic prostaglandin E synthase (PGES) protein were reported in schizophrenia compared with healthy controls. In schizophrenia, cytosolic PGES was elevated in the prefrontal cortex, but no changes were observed in the temporal and occipital cortices.^140^ COX-1 and 2, enzymes regulating the production of prostaglandin E_2_, were not altered in the brains of schizophrenics.^140^ No changes in COX-2 mRNA expression were also observed in the dorsolateral prefrontal cortex^86, 141^ and middle frontal gyrus,^139^ whereas COX-1 mRNA expression was unchanged in the dorsolateral prefrontal cortex.^141^ Similarly, immunohistochemical analysis of the hippocampus shows no differences in COX-2-positive cell density between schizophrenia and healthy controls.^142^ It should be noted that age did affect COX-1 and COX-2 mRNA expression in schizophrenia, with older schizophrenia patients having increased COX-1 and decreased COX-2 mRNA expression.^141^ ALOX5AP, a protein regulating 5-lypoxygenase (LOX) activity, was found to have lower mRNA expression in the temporal lobe of 66 schizophrenia patients compared with control patients.^98^

In contrast, Rao *et al.* observed no changes in cytosolic PGES mRNA and protein in the frontal cortex in schizophrenia. They also reported no changes in other arachidonic cascade enzymes, such as calcium-independent phospholipase (PLA)2, LOX5, LOX12, LOX15 and microsomal PGES. They did, however, find COX-2 to be increased in schizophrenia, along with cPLA_2_ and sPLA_2_.^73^

## Substance P

Substance P has been measured in postmortem brains of patients with schizophrenia in 11 studies (Table 6).

One study evaluated preprotachykinin A, a precursor to substance P, and reported that mRNA measured by *in situ* hybridization is decreased in the basal and lateral nuclei of the amygdala, whereas no changes were measured in the temporal cortex.^143^ Similarly, the density of cells containing preprotachykinin A mRNA measured by *in situ* hybridization is also not changed in the caudate and putamen in schizophrenia.^144^ Substance P density in multiple brain regions, including substantia nigra,^145^ caudate nucleus,^146^ frontal cortex,^146^ basal ganglia^146^ and hypothalamus,^146^ detected by radioimmunoassay, is not different in schizophrenia compared with healthy controls. Psychosis without schizophrenia, such as affective disorder and unspecified functional psychosis, did exhibit higher substance P protein concentrations.^146^ An immunohistochemical study also did not detect any changes in substance P in the basal ganglia of six schizophrenia patients compared with unaffected controls.^147^

Two studies, however, have reported differences in substance P concentration in schizophrenia. Toru *et al.*^148^ found a significant increase in substance P detected by radioimmunoassay in the orbitofrontal cortex and hippocampus, and in antipsychotic medication users in the thalamus, substantia nigra and temporal cortex.^148^ Similarly, Roberts *et al.* found increased hippocampal substance P, but no changes were seen in multiple brain regions including the amygdala, thalamus, basal ganglia, and temporal, frontal, parietal and cingulate cortices.^149^

Five studies evaluated substance P binding to substance P neurokinin 1 receptor. Autoradiography found no changes in neurokinin 1 receptor density in the putamen,^150^ anterior cingulate cortex^151^ and temporal cortex.^143^ There was, however, an increase in receptor density in the caudate^150^ and nucleus accumbens.^150^ Immunohistochemical analysis found similar increases in substance P receptor in the prefrontal cortex in schizophrenia,^152^ but not in the amygdala.^153^ This lack of change in the amygdala cell density expressing substance P receptor was consistent with mRNA expression.^153^

## Other markers

Multiple other markers associated with inflammation that do not fit the categories mentioned above have also been measured in postmortem brains of scizophrenic patients to evaluate a potential link between neuroinflammation and schizophrenia. We identified 16 studies evaluating miscellaneous markers in postmortem brains in schizophrenia (Table 7).

ICAM-1 is a marker of neuroinflammation, associated with blood–brain barrier disruption. Thomas *et al.*^154^ found no differences in ICAM-1 labeled cells in both the dorsolateral prefrontal cortex and anterior cingulate cortex of 15 schizophrenia patients of the Stanley Foundation Neuropathology Consortium compared to healthy controls.

Four studies investigated the NF-κB pathway in postmortem schizophrenic brains. Rao *et al.*^73^ measured increases in both NF-κB p50 and p65 subunits mRNA expression in the BA10 of schizophrenia patients. A second study evaluating the prefrontal cortex of schizophrenics reported increased NF-κB1 and 2 mRNA expression.^155^ However, 2 separate studies could not detect any differences in NF-κB2 expression in the frontal cortex^156^ and NF-κB in the dorsolateral prefrontal cortex^86^ between schizophrenics and healthy controls. Schnurri-2, a NF-κB site binding protein inhibiting downstream transcription, has been reported to be decreased in the prefrontal cortex of schizophrenia patients.^155^

Microarray analyses followed by qPCR have proposed markers associated with the immune system or inflammatory response being associated with schizophrenia. One such marker, which was reported in four microarray analyses, is SERPINA3, a protease inhibitor that is involved in inflammatory processes and connective tissue turnover. In the dorsolateral prefrontal cortex, SERPINA3 mRNA expression was significantly higher in the brains of schizophrenics compared with healthy controls.^86^ The same group confirmed this finding in a second cohort, finding increased SERPINA3 mRNA expression in the medial frontal gyrus in schizophrenia, whereas changes in IL-1RL1 expression were not detected.^139^ Similar increases of SERPINA3 mRNA expression were reported in two other microarray studies in the frontal cortices of 55 (ref. 93) and 14 (ref. 157) schizophrenia patients and were confirmed by qPCR.

These two microarray studies also found elevated interferon-induced transmembrane protein (IFITM)1, 2 and 3, proteins involved in regulation of the immune response, mRNA expression in the prefrontal cortex in schizophrenia.^93, 157^ A third study confirmed the increased IFITM3 mRNA expression in the prefrontal cortex.^158^ Similar overexpression of IFITM1, 2 and 3 was observed by microarray and confirmed by qPCR in the hippocampus of schizophrenic patients.^77^ A fifth study targeted IFITM1 and 2/3 expression in a separate cohort of prefrontal cortices of schizophrenia patients and reported an increase in both markers independent of antipsychotic use.^159^

Other markers that either increased or decreased in microarrays include CD163 and S100a8 and 9 in the hippocampus,^77^ CHI3L1 (ref. 157) and GBP1 (ref. 93) in the prefrontal cortex, TNFSF8, 10 and 13 (although 8 and 13 were not significant in PCR validation) in the dorsolateral prefrontal cortex,^160, 161^ and TIMP1, TYROB and TNFSRF1A in the temporal lobe.^98^ However, unlike the decrease in TIMP1 mRNA expression measured in the temporal lobe, TIMP1 protein concentration, measured by enzyme-linked immunosorbent assay, was not changed in the prefrontal cortex in another study.^137^ Schmitt *et al.* reported 6 out of 23 immune-related genes are downregulated in the superior temporal cortex in schizophrenia. The 23 immune-related genes include cytokines and microglial markers, discussed above, and other markers including LPL, CFD, PTGER4 and EDG3 being downregulated and ITGA1, LCP1, LTC4S, MTHFD2, CD84, GPX, IFI16 and SOD2 being unchanged.^94^

## Discussion

Schizophrenia has been linked to neuroinflammation.^8, 9, 10^ Schizophrenic patients have been shown to have elevated cytokines in blood^17, 18^ and elevated microglia activation in the brain as measured by PET analysis in some^22, 23^ but not all^25, 26^ reports. This paper systematically reviewed the literature covering neuroinflammatory analyses in postmortem brains from schizophrenic patients.

Multiple studies evaluating neuroinflammation in postmortem brain samples found evidence of neuroinflammation in schizophrenia. However, a definitive statement cannot be made on whether neuroinflammation is present in schizophrenic postmortem brain samples due to the large number of null studies. For example, out of 33 studies evaluating GFAP, 21 studies did not find any effect of schizophrenia on GFAP expression, whereas 6 studies found a decrease in GFAP and 6 studies had elevated GFAP expression. Similarly, out of 34 studies that evaluated glial cell density, 25 studies found no effect of schizophrenia, whereas 7 studies found a decrease in glial cells and 2 studies found an increase. Variability is also observed for four microglial markers (HLA, CD11b, CD68 and calprotectin), where 11 studies had elevated expression of microglial markers, 8 studies found no differences and 3 found a decrease. SERPINA3, a protease inhibitor that is involved in inflammatory processes and connective tissue turnover, however, was elevated in the 4 studies, which have reported on its mRNA expression. IFITM, a viral restriction factor, was also reported elevated in four microarrays, and confirmed in one targeted study.

These discrepancies may be explained, at least partly, by the heterogeneity in study designs across studies. One of the heterogeneous variable across studies is brain region analyzed. For example, studies evaluating GFAP expression have analyzed 34 brain regions, including the hippocampus, prefrontal cortex, enthorhinal cortex, orbitofrontal cortex and cingulate cortex among others. Whereas all five studies analyzing GFAP expression in the entorhinal cortex found no differences in schizophrenia, 4 of the 13 studies evaluating GFAP expression in the frontal cortex, prefrontal cortex or dorsolateral prefrontal cortex (BA9, 10 or 46) identified differences between schizophrenia and healthy controls. However, classification of the frontal cortices varied between studies and may explain differing results. Moreover, four out of six studies examining the cingulate cortex, subgenual cingulate cortex or anterior cingulate cortex found significant changes in GFAP in schizophrenia. It is possible that certain brain regions, such as the cingulate cortex, are more susceptible to change in schizophrenia compared to other regions such as the entorhinal cortex. Nevertheless, despite more studies pointing to a decrease in GFAP expression in the cingulate cortex in schizophrenia, not all studies show decreases despite evaluating the same brain region and marker.^53, 58^

Consideration of the cortical layer in which the markers are measured may be needed in order to tease out the differences across studies. Many studies found layer-specific effects in various brain regions and markers. For example, in two studies, GFAP expression was increased solely in layer V of the dorsolateral prefrontal cortex^70^ and layer I in subgenual cingulate cortex.^67^ This could explain differences across studies measuring GFAP in the whole prefrontal cortex mentioned above. Similarly, layer-specific effects of schizophrenia on glial cell density measured by cresyl violet were observed in several studies evaluating the motor cortex (layer III), planum temporale (layer IV), cingulate cortex (layer IV) and dorsolateral prefrontal cortex (layer V).

Differences in methodological approaches also warrant consideration when evaluating the results of the studies mentioned above. Stereological analysis, an unbiased cell counting method, was applied to approximately half of the studies measuring glial cells. Only one study utilizing stereology measured differences in glial cell density, whereas seven studies using other methods reported differences. However, the use of stereology is not always clear in the methods section and therefore the results above should be considered with caution. Similarly, double labeling could be utilized to detect different subtypes of cells. However, few studies in this review utilized double labeling, which should be considered when no differences in cell densities are detected. Thus, the lack of changes in cell densities may not reflect changes in subtypes of cells.

Another variable that may contribute to the heterogeneous results is the stage of the disorder. By separating paranoid schizophrenia from residual schizophrenia, differences in S100b-positive cells were observed.^79^ Microglia are also elevated in paranoid schizophrenia, where HLA-DR-positive cell density is higher in paranoid schizophrenia compared with residual schizophrenia.^88^ Moreover, differences in gliosis score are seen between early onset and late onset schizophrenia.^135^ Similarly, the three patients with microgliosis in the study by Bayer *et al.*^85^ were all defined to have late onset schizophrenia.

Suicide is common in schizophrenia. This is important to consider as postmortem brains from suicide victims may present elevated pro-inflammatory cytokines.^162, 163^ This is in agreement with Steiner and colleagues where the two schizophrenia patients that committed suicide had the highest HLA-DR-positive cell density.^91^ When accounting for suicide victims, the same group found no differences between diagnosis groups. They did, however, find a relation between suicide and HLA-DR-positive cells.^92^ Similarly, GFAP cell density is elevated in the dorsolateral prefrontal cortex of suicide victims compared with non-suicide schizophrenic patients.^55^ This effect on GFAP, in the dorsolateral prefrontal cortex of suicide victims, however, was not seen in another study measuring GFAP by western blot.^65^ No effect of suicide was also observed for ICAM-1 expression.^154^ This is also an important consideration for control group selection. Tooney *et al.*^152^ found an effect of schizophrenia on neurokinin 1 receptor compared with a control group that contained suicide victims, which may potentially confound the results. Although a few studies considered the effect of suicide on their measurements, many studies do not report this data or include it in their statistical analysis, making it a limitation and should be considered in future studies.

Several other confounding factors have been associated with potential effects on neuroinflammatory markers in schizophrenia in postmortem brains. Antipsychotics have been associated with modulation of inflammation.^164^ Typical antipsychotics generally reduce pro-inflammatory markers while atypical antipsychotics generally increase them.^164, 165^ In our systematic review, antipsychotics were reported to raise GFAP,^53, 71, 74^ substance P^148^ and HLA.^53^ No effect of medication, however, was seen on IL-1β.^136^ This is important to note, as not all studies measured antipsychotic levels at time of death or corrected for this potential confounder. Moreover, even when measured, separation of typical and atypical antipsychotics was not considered in the statistical analysis. Also, control subjects would not have been exposed to antipsychotic medication, potentially creating a confounder between controls and the experimental group. Similarly, age is positively correlated to the expression of GFAP,^66^ S100^58^ and substance P receptor binding.^151^ Lifestyle choices, such as smoking and alcohol abuse, may also contribute to neuroinflammation. In one study, decreases in MHC I observed in the dorsolateral prefrontal cortex of non-smoking schizophrenia patients were no longer apparent in the smoking population.^100^ Interestingly, lifestyle choices and antipsychotic use are also risk factors for the development of type II diabetes,^166^ which is more prevalent in schizophrenia^167^ and has been associated with neuroinflammation.^168, 169^ Although not reported in the studies in this review, it would be of interest for future studies to investigate a potential link between diabetes in schizophrenia and neuroinflammation.

The source of the brains also needs consideration. Several brain banks produced multiple studies utilizing several different brain regions from the same brains. Brain banks may have different diagnosis methods, inclusion and exclusion criteria, storage, and demographics among many other variables. Thus, it is possible that the results may be biased by where the brains samples used were provided from. For example, the 33 studies on GFAP reported in this paper were generated from brains from 15 separate brain banks. Of those 33 studies, 6 studies reported a decrease in GFAP. Of those 6 studies, 2 studies utilized the Stanley Foundation Neuropathology Consortium whereas 3 other studies used the Corsellis Brain Collection.

Despite the heterogeneity across studies, the expression of both SERPINA3 and IFITM was repeatedly found to be increased in microarray studies. SERPINA3, a member of the serine protein inhibitor family, is an acute-phase protein which increases during inflammatory episodes^170^ and is expressed in reactive astrocytes.^171^ SERPINA3 has previously been linked with decreased age of onset of Alzheimer's symptoms.^172^ Moreover, SERPINA3 expression is correlated with GFAP positive cells in Alzheimer's disease.^173^ Patients with multiple sclerosis have elevated SERPINA3 CFS concentration.^174^ In depression, no association was reported between blood levels of SERPINA3 and symptoms.^175^ IFITM, on the other hand, is an immune-related protein involved in viral replication. In animal models of inflammation, IFITM1 is increased in the cortex of mice lacking the NF-κB site binding protein Schnurri-2.^176^ Similarly, IFITM1 and 3 expression is upregulated in the hippocampus following centrally administered lipopolysaccharide injection,^177^ suggesting its involvement in neuroinflammatory processes.

In conclusion, although the majority of studies note a lack of change in neuroinflammatory markers in postmortem brain samples of patients with schizophrenia, there are still multiple studies indicating either increases or decreases in neuroinflammatory markers. Although ~70% of studies evaluating astrocytes or glial cells in schizophrenia found no change, there were still ~30% of studies showing either an increase or decrease in astrocytic markers and glial cell density. The changes in microglial markers in schizophrenia is more variable across studies, with ~45% of studies showing an increase and 40% of studies showing no change. Similarly, pro-inflammatory cytokine concentration in the postmortem schizophrenia brain is also variable across studies, with studies showing both elevated and decreased cytokine levels in schizophrenia. The cause of this heterogeneity in results is not clear at the moment, but may be due to several factors including brain region measured, stage of disorder, source of the brain and medication. Despite this heterogeneity, microarray analyses have consistently indicated markers such as SERPINA3 and IFITM to be elevated in schizophrenia. Future studies should consider these potential sources of heterogeneity when measuring neuroinflammatory markers in postmortem brain samples of schizophrenia patients.

## Figures and Tables

**Table 1:** Astrocytes in postmortem schizophrenia brain

*Author*	*Brain bank*	n	*Sex (m/f)*	*Age*	*Death from suicide*	*Brain region*	*Technique*	*Inflammatory markers*	*Results*
Altshuler *et al.*^48^	SFNC	scz 9 ctr 14	scz 5/4 ctr 8/6	scz 45 ctr 47	scz 3	Basolateral nucleus of the amygdala	IHC	GFAP	↔
Arnold *et al.*^45^	Prospective study	scz 7* scz+d 14# ctr 12	scz 3/4 scz+d 6/8 ctr 5/7	scz 74 scz+d 82 ctr 75	NA	EC*, SB#, CA3*, CA1*, DG*, MFC*, OFC#, VC*	IHC	GFAP#, VIM*	↔*↑#
Arnold *et al.*^46^	Prospective study	scz 23 ctr 14	scz 8/15 ctr 6/8	scz 80 ctr 75	NA	EC (BA 28) CA1 HPC, SB MFC (BA9 and 46), OFC (BA11), CL (BA17)	IHC	GFAP	↔
Barley *et al.*^74^	SFNC	Varies across brain regions	Varies across brain regions	Varies across brain regions	NA	AVN, PU, IC, MTN	PCR	GFAP, ALDH1	↑
Beasley *et al.*^59^	NYSPIBC	scz 15 ctr 13	scz 9/6 ctr 10/3	scz 54 ctr 51	None	Anterior limb of internal capsule	ELISA	GFAP	↔
Casanova *et al.*^80^	NC	scz 6 ctr 7	scz 4/2 ctr 4/3	scz 39 ctr 61	NA	DG, PP	Holzer's Technique	Astrocytes	↔
Catts *et al.*^75^	NSWTRC	scz 37 ctr 37	scz 24/13 ctr 30/7	scz 51 ctr 51	scz 8	DLPFC (BA46)	PCR, IHC, WB	GFAP	↔
Damadzic *et al.*^54^	(1) CBDBNIMH (2) SFNC	Study (1) scz 7 ctr 8 study (2) scz 14 ctr 15	Study (1) scz 3/4 ctr 3/5 study (2) scz 9/5 ctr 9/6	Study 1 scz 49 ctr 47 study 2 scz 46 ctr 48	Study 1 scz 3 ctr 1 study 2 scz 3	EC	IHC	GFAP	↔
Dean *et al.*^62^	NA	scz 20 ctr 20	scz 13/7 ctr 13/7	scz 56 ctr 56	NA	BA9, 10, 40, 46	WB, PCR	S100b, GFAP	↔
Falkai *et al.*^49^	DBC	scz 33 ctr 26	scz 14/19 ctr 13/13	scz 54 ctr 53	scz 4	PMC, SB, EC, IH, SVZ3V	IHC	GFAP	↔
Falke *et al.*^50^	Prospective study	scz 12 ctr 11	scz 3/9 ctr 7/4	Scz 81 ctr 78	NA	MTN, CT	IHC	GFAP	↔
Fatemi *et al.*^60^	SFNC	scz 15 ctr 15	scz 9/6 ctr 9/6	scz 44 ctr 48	scz 4	lateral CB	WB	GFAP	↔
Feresten *et al.*^65^	SMRIAC	scz 35 ctr 35	scz 26/9 ctr 26/9	scz 43 ctr 44	scz 7	DLPFC (BA9)	WB	GFAP#, VIM*, ALDH1L1*, EAAT*	↔* ↑#
Hercher *et al.*^55^	SMRIAC	scz 20 ctr 20	scz 13/7 ctr 14/6	scz 45 ctr 45	scz 4	DLPFC (BA9)	IHC	GFAP	↔
Hwang *et al.*^77^	SFNC, SMRIAC	scz 33 ctr 34	scz 23/10 ctr 23/11	scz 44 ctr 46	NA	HPC	PCR, IHC	APOL1, ADORA2A	↑
Karson *et al.*^61^	DCMEO	scz 25 ctr 28	scz 22/3 ctr 22/6	scz 34 ctr 35	scz 16 ctr 8	FC, TC, OC, CB, TH, pons	WB	GFAP	↔
Karson *et al.*^63^	NA	scz 14 ctr 12	scz 13/1 ctr 11/1	scz 65 ctr 67	NA	PFC (BA10)	WB, northern blot	GFAP	↔
Katsel *et al.*^58^	NA	scz 18 ctr 21	scz 10/8 ctr 10/11	scz 78 ctr 77	scz 0	Cingulate cortex (BA24/32)	PCR	GFAP #, S100b*, VIM#, EAAT2*, ALDH1L1#, AQP4*, DIO*, GS*, THBS4*, GL#	↓*↔# (deep layer only)
Kolomeets *et al.*^83^	ADMPH	scz 19 ctr 16	scz 11/8 ctr 11/5	scz 54 ctr 56	NA	HPC	Electron microscope	Astrocytes	↔
Markova *et al.*^72^	NA	scz 12 ctr 10	NA	scz 62 ctr NA	NA	Olfactory tubercle	IHC	GFAP	↑
Pakkenberg^82^	NA	scz 12 ctr 12	scz 8/4 ctr 66	scz 63 ctr 62	NA	MTN*, AG#, NAS*, PL#	Nissl	Astrocytes	↓*↔#
Pantazopoulos *et al.*^47^	HBTRC	scz 11 ctr 15	scz 7/4 ctr 10/5	scz 62 ctr 66	scz 1	AG, EC	IHC	GFAP	↔
Perrone-Bizzozero *et al.*^64^	HBTRC	scz 17 ctr 18	scz 17/0 ctr 18/0	scz 44 ctr 48	scz 4 ctr 2	VC (BA17,20) PFC (BA9,10)	WB	GFAP	↔
Radewicz *et al.*^53^	Prospective study	scz 12 ctr 11	NA	scz 80 ctr 72	NA	DLPFC (BA9), ACC (BA24), superior TC (BA22)	IHC	GFAP	↔
Rajkowska *et al.*^70^	CCCO	scz 9 ctr 15	scz 2/7 ctr 10/5	scz 47 ctr 47	scz 3	DLPFC (BA9)	IHC	GFAP	↑ (layer V only)
Rao *et al.*^73^	HBTRC	scz 10 ctr 10	scz 6/4 ctr 7/3	scz 59 ctr 49	NA	FC (BA10)	IHC, PCR, WB	GFAP	↑
Roberts *et al.*^43^	VIBR	scz 5 ctr 7	scz 1/4 ctr 4/3	scz 39 ctr 51	NA	TL, PC, PU, CT, AG, HPC, TH	IHC	GFAP	↔
Roberts *et al.*^44^	Runwell series 1 brain collection	scz 18 ctr 12	scz 14/4 ctr 9/3	scz 69. ctr 55	NA	TL	IHC	GFAP	↔
Schmitt *et al.*^81^	DBC	scz 10 ctr 10	scz 5/5 ctr 5/5	scz 55 ctr 50	scz 1	CA1,2/3,4, SB	Cresyl violet	Astrocytes	↔
Steffek *et al.*^66^	MSMC, BVAMC	scz 23 ctr 27	scz 16/7 ctr 14/13	scz 72 ctr 79	None	DLPFC#, VC#, ACC*, HPC#, temporal gyrus#	WB	GFAP	↓*↔#
Steiner *et al.*^79^	MBC	p scz 9* r scz 9# ctr 16	p scz 5/4 r scz 4/5 ctr 7/9	p scz 56 r scz 54 ctr 56	p scz 3 r scz 2	ACC*, DLPFC#, OFC #, sTC#, HPC#, MTN#	IHC	S100b	↑*↔#
Steiner *et al.*^78^	WMSH, HUIN	scz 9 ctr 7	scz 5/4 ctr 5/2	scz 68 ctr 65	None	CO	WB, MS	S100b	↓
Stevens *et al.*^51^	VIBR	scz 5 ctr 7	NA	NA	NA	CT, PVN	IHC	GFAP	↔
Tkachev *et al.*^57^	SFNC	scz 15 ctr 15	scz 9/6 ctr 9/6	scz 44 ctr 48	scz 4	PFC (BA9)	PCR	GFAP	↔
Toro *et al.*^71^	SFNC	scz 15 ctr 15	scz 9/6 ctr 9/6	scz 45 ctr 48	scz 4	PFC* (BA9,32,46) OFC# (BA11/47)	IAR	GFAP	↑*↓#
Uranova *et al.*^84^	MHRC	scz 26 ctr 26	scz 11/15 ctr 21/5	scz 53 ctr 52	NA	PFC (BA10) and VC (BA17)	Electron microscopy	Astrocytic end feet	↑ (except for VC of non p scz)
Williams *et al.*^67^	CC	scz 10 ctr 19	scz 5/5 ctr 11/8	scz 58 ctr 66	scz 1	Subgenual cingulate cortex*, CO	IHC	GFAP	↓ (only in layer I*)
Williams *et al.*^52^	CC	scz 13 ctr 16	Scz 6/7 ctr 4/12	scz 57 ctr 55	scz 2	Nucleus basalis	IHC	GFAP	↔
Williams *et al.*^69^	CC	scz 12 ctr 13	scz 7/4 ctr 9/4	scz 60 ctr 52	scz 2	Substantia nigra	IHC	GFAP	↓
Williams *et al.*^68^	CC	scz 10 ctr 19	scz 5/5 ctr 11/8	scz 58 ctr 66	scz 1	Subgenual cingulate cortex	IHC	GFAP	↓
Webster *et al.*^56^	SFNC	scz 15 ctr 15	scz 9/6 ctr 9/6	scz 44 ctr 48	scz 4	DLPFC, HPC	IHC	Phosphorylated GFAP	↔ (except DLPFC blood vessel labeling)
Webster *et al.*^76^	SFNC	scz 15 ctr 15	scz 9/6 ctr 9/6	scz 45 ctr 48	scz 4	ACC (BA24)	Riboprobe and *in situ* hybridization	GFAP	↓ (white matter only)

Abbreviations: ACC, anterior cingulate cortex; ADMPH, Anatomical Department of Moscow Psychiatric Hospital; ADORA2A, adenosine A2A receptor; AG, amygdala; ALDH, aldehyde dehydrogenase; APOL, apolipoprotein; AQP, aquaporin; AVN, anteroventral nucleus; BA, Brodmann area; BVAMC, Bronx Veterans Administration Medical Center; CA, cornu ammonis; CB, cerebellum; CBDBNIMH, Clinical Brain Disorder Branch at the National Institute of Mental Health; CC, Corsellis Collection; CL, calcarine cortex; CO, corpus callosum; CT; caudate; ctr, control; CCCO, Cuyahoga Country Coroner's Office; d, dementia; DBC; Dusseldorf Brain Collection, DCMEO, District of Columbia Medical Examiner's Office; DG, dentate gyrus; DIO, diodinase; DLFPC, dorsolateral prefrontal cortex; EAAT, excitatory amino-acid transporter; EC, entorhinal cortex; ELISA, enzyme-linked immunoadsorbent assay; FC, frontal cortex; GFAP, glial fibrillary acidic protein; GL, phosphate-activated glutaminase; GS, glutamine synthase; HBTRC, Harvard Brain Tissue Resource Centre; HPC, hippocampus; HUIN, Heidelberg University Institute of Neuropatholagy; IAR, immunoautoradiography; IC, internal capsule; IH, inferior horn; IHC, immunohistochemistry; MBC, Magdeburg Brain Collection; MHRC, Mental Health Research Centre; MFC, midfrontal cortex; MS, mass spectrometry; MSMC, Mount Sinai Medical Centre; MTN, mediodorsal thalamic nucleus; NA, not available; NC, Neuman Collection; NSWTRC, New South Wales Tissue Resource Centre; NYSPIBC, New York State Psychiatric Institute Brain Collection; OC, occipital cortex; OFC, orbitofrontal cortex; PC, parietal cortex; PCR, polymerase chain reaction; PFC, prefrontal cortex; PMC, premotor cortex; PP, perforant path; PU, putamen; PVN, paraventricular nucleus; SB, subiculum scz, schizophrenia; scz (p), paranoid schizophrenia; scz (r), residual schizophrenia; SFNC, Stanley Foundation Neuropathology Consortium; SMRIAC, Stanley Medical Research Institute Array Collection; ST, striatum; SVZ, subventricular zone; TC, temporal cortex; TH, thalamus; THBS, thrombospondin; TL, Temporal lobe; VBBN, Victorian Brain Bank Network; VC, visual cortex; VIBR; Vogt Institute of Brain Research; VIM, vimentin; WB, western blot; WMSH, Wiesloch Mental State Hospital.

*, # indicate which variables results are representing.

**Table 2:** Microglia in postmortem schizophrenia brain

*Author*	*Brain bank*	n	*Sex (m/f)*	*Age*	*Death from suicide*	*Brain region*	*Technique*	*Inflammatory markers*	*Results*
Arnold *et al.*^46^	Prospective study	scz 23 ctr 14	scz 8/15 ctr 6/8	scz 80 ctr 75	NA	EC (BA 28) CA1, SB, MFC (BA9 and 46), OFC (BA11), CL (BA17)	IHC	CD68	↔
Bayer *et al.*^85^	INUBMC, INMD	scz 14 ctr 13	scz 3/11 ctr 8/5	scz 64 ctr 58	NA	FC, HPC	IHC	HLA-DR	↑
Busse *et al.*^88^	MBC	scz (p) 10* scz (r) 7 ctr 11	scz (p) 5/5 scz (r) 4/3 ctr 6/5	scz (p) 50 scz (r) 56 ctr 56	scz 5#	HPC	IHC	HLA-DR	↑*#
Comte *et al.*^95^	SFNC	Scz 15 ctr 15	scz 9/6 ctr 9/6	scz 44 ctr 48	scz 4	SVZ	IHC	MHC II	↔
Connor *et al.*^97^	HBTRC	scz 22 ctr 45	scz 9/13 ctr 24/21	scz 68 ctr 70	NA	ACC (BA24), DLPFC	IHC	Iba1	↔
Durrenberger *et al.*^98^	BBPDGU	scz 10 ctr 10	scz 5/5 ctr 5/5	scz 66 ctr 61	NA	Temporal lobe (BA22)	PCR	HLA-DRA, HLA-DRB4	↓
Falke *et al.*^50^	Prospective study	scz 12 ctr 11	scz 3/9 ctr 7/4	scz 81 ctr 78	NA	MTN, CT	IHC	CD68	↔
Fillman *et al.*^86^	NSWTRC	scz 37 ctr 37	scz 24/13 ctr 30/7	scz 53 ctr 51	NA	DLPFC (BA46)	WB, IHC	HLA-DR/DP/DQ	↑
Foster *et al.*^90^	SFNC	scz 15 ctr 15	scz 9/6 ctr 9/6	scz 44 ctr 48	scz 4	DLPFC (BA9)	ELISA, IHC	Calprotectin* CD68#	↑*↔#
Gos *et al.*^99^	MBC	scz 13 ctr 12	scz 7/6 ctr 6/6	scz 51 ctr 49	scz 2	CA1*,2,3, DG	IHC	HLA-DR#, quinolinic acid*	↓*↔#
Hercher *et al.*^55^	SMRIAC	scz 20 ctr 20	scz 13/7 ctr 14/6	scz 45 ctr 45.3	scz 4	DLPFC (BA9)	IHC	Iba1	↔
Kano *et al.*^100^	SMRIAC	scz 35 ctr 35	scz 26/9 ctr 26/9	scz 43 ctr 44	scz 7	DLFPC*, OFC#	WB	MHC I	↓*↔#
Nakatani *et al.*^96^	VIFM	scz 7 ctr 7	scz 3/4 ctr 3/4	scz 61 ctr 61	scz 1	DLPFC (BA46), PC (BA40)	PCR	HLA-DRA	↔
Radewicz *et al.*^53^	Prospective study	scz 12 ctr 11	NA	scz 80 ctr 72	NA	DLPFC (BA9)*, ACC (BA24)#, superior TC (BA22)*	IHC	HLA-DR	↑*↔#
Rao *et al.*^73^	HBTRC	scz 10 ctr 10	scz 6/4 ctr 7/3	scz 59 ctr 49	NA	FC (BA10)	IHC, PCR, WB	HLA-DR, CD11b	↑
Saetre *et al.*^93^	SFNC, HBTRC, MBB	scz 55 ctr 55	NA	scz 58 ctr 56	NA	FC (BA8 and 9), superior frontal gyrus	PCR	HLA-A	↔
Schmitt *et al.*^94^	BBPDGU	10 per group	scz 5/5 ctr 8/2	scz 66 ctr 61	NA	TC (BA22)	PCR	HLA-DRB3, HLA-DPA1	↔
Sinkus *et al.*^101^	SRCBB	scz 42 ctr 47	scz 28/14 ctr 33/14	scz 51 ctr 53	scz 0 ctr 1	HPC	PCR	HLA-A#, HLA-B*	↑*↔#
Steiner et al.^91^	MBC	scz 16* ctr 16	scz 8/8 ctr 8/8	scz 55 ctr 58	scz 2#	HPC, ACC, DLPFC, MTN	IHC	HLA-DR	↔*↑#
Steiner *et al.*^92^	MBC	scz 16* ctr 10	scz 7/9 ctr 5/5	scz 54 ctr 55	scz 6#	HPC*, DLPFC#, ACC#, MTN#	IHC	HLA-DR	↔*↑#
Wierzba-Bobrowicz *et al.*^89^	NA	scz 12 ctr 7	scz 0/12 ctr 0/6	scz 59 ctr 56	NA	Frontal lobe, cingulate gyrus (BA24)	IHC	HLA-DP/DQ/DR	↑
Wierzba-Bobrowicz *et al.*^87^	NA	scz 9 ctr 6	NA	scz 56 ctr 56	NA	Gyrus temporal inferior (BA20), gyrus cinguli (BA24)	IHC	HLA-DP/DQ/DR	↑

Abbreviations: ACC, anterior cingulate cortex; BA, Brodmann area; BBPDGU, Brain Bank for Psychiatric Diseases at the Gottingen University; CA, cornu ammonis; CD, cluster of differentiation; CL, calcarine cortex; CT, caudate; ctr, control; DG, dentate gyrus; DLFPC, dorsolateral prefrontal cortex; EC, entorhinal cortex; ELISA; enzyme-linked immunoadsorbent assay; FC, frontal cortex; HBTRC, Harvard Brain Tissue Resource Centre; HLA, Human Leukocyte Antigen; HPC, hippocampus; IHC, immunohistochemistry; Iba, ionized calcium-binding adaptor molecule; INMD, Institute for Nervous and Mental Diseases; INUBMC, Institute of Neuropathology, University of Bonn Medical Centre; MBB, Maudsley Brain Bank; MBC, Magdeburg Brain Collection; MHC, major histocompatibility complex; MFC, midfrontal cortex; MTN, mediodorsal thalamic nucleus; NA, not available; NSWTRC, New South Wales Tissue Resource Centre; OFC, orbitofrontal cortex; PC, parietal cortex; PCR, polymerase chain reaction; SB, subiculum; scz, schizophrenia; scz (p), paranoid schizophrenia, scz (r). residual schizophrenia; SFNC, Stanley Foundation Neuropathology Consortium; SMRIAC, Stanley Medical Research Institute Array Collection; SRCBB, Schizophrenia Research Center Brain Bank; SVZ, subventricular zone; TC, temporal cortex; VIFM, Victorian Institute of Forensic Medicine; WB, western blot.

*, # indicate which variables results are representing.

**Table 3:** Undifferentiated glial cells and postmortem schizophrenia brain

*Author*	*Brain bank*	n	*Sex (m/f)*	*Age*	*Death from suicide*	*Brain region*	*Technique*	*Inflammatory markers*	*Results*
Beasley *et al.*^129^	SFNC	scz 15 ctr 15	scz 9/6 ctr 9/6	scz 44 ctr 48	scz 4	Planum temporal	Cresyl violet	Glia	↔
Beasley *et al.*^107^	SFNC	scz 15 ctr 15	scz 9/6 ctr 9/6	scz 44 ctr 48	scz 4	Planum temporal	Cresyl violet	Glia	↓
Beckmann and Lauer^133^	WBC	scz 9 ctr 9	scz 9/0 ctr 9/0	scz 55 ctr 52	scz 1	ST, PU, NAS, CT	Gallocyanin	Glia	↔
Benes *et al.*^110^	HBTRC	scz 10 ctr 10	NA	scz 60 ctr 66	scz 1	PFC (BA10)#, motor cortex (BA4)*, cingulate cortex (BA24)#	Cresyl violet	Glia	↓* (only layer III) ↔#
Benes *et al.*^125^	HBTRC	scz 9 scz+md 9 ctr 12	NA	scz 53 scz+md 49 ctr 59	NA	PFC (BA10), ACC (BA24)	Cresyl violet	Glia	↔
Benes *et al.*^123^	HBTRC	scz 11 ctr 12	scz 7/4 ctr 7/5	scz 52 ctr 58	scz 5	ACC (BA24)	Cresyl violet	Glia	↔
Bezchlibnyk *et al.*^112^	SFNC	scz 13 ctr 15	scz 8/5 ctr 9/6	scz 47 ctr 48	NA	AG	Nissl	Glia	↔
Bogerts *et al.*^130^	VIBR	scz 6 ctr 9	scz 2/6 ctr 5/4	scz 51 scz 43	NA	SN	Cresyl violet	Glia	↔ (reduction in size)
Brauch *et al.*^106^	SNFC	scz 13 ctr 14	NA	scz 46 ctr 47	NA	TC	Cresyl violet	Glia	↓
Bruton *et al.*^103^	NA	scz 48 ctr 56	NA	NA	NA	FC, PC, TC	Holzer's Technique	Glia	↑
Chana *et al.*^124^	SFNC	scz 15 ctr 15	scz 9/6 ctr 9/6	scz 45 ctr 48	scz 7	ACC (BA24)	Cresyl violet	Glia	↔
Chana *et al.*^121^	SFNC	scz 14 ctr 15	NA	NA	NA	MTN	NA	Glia	↔
Cotter *et al.*^109^	SFNC	scz 15 ctr 15	scz 9/6 ctr 9/6	scz 45 ctr 48	scz 7	ACC	Cresyl violet	Glia	↔
Cotter *et al.*^108^	SFNC	scz 15 ctr 15	scz 9/6 ctr 9/6	scz 45 ctr 48	scz 7	DLFPC (BA9, 46)	Cresyl violet	Glia	↓ (only layer V)
Cotter *et al.*^122^	SFNC	scz 15 ctr 15	scz 9/6 ctr 9/6	scz 44 ctr 48	scz 4	Heschl's gyrus (BA41) (layer 3 and 5)	Cresyl violet	Glia	↔
Cotter *et al.*^127^	SFNC	scz 15 ctr 15	scz 9/6 ctr 9/6	scz 45 ctr 48	scz 7	OFC	Cresyl violet	Glia	↔
Crow *et al.*^134^	NA	scz 22 ctr 26	NA	NA	NA	Temporal horn	Holzer's Technique, IHC	Glia, diazepam binding inhibitor-like	↔
Cullen *et al.*^120^	NA	scz 10 ctr 10	scz 6/4 ctr 6/4	scz 60 ctr 60	NA	Frontal gyrus (BA9)	Cresyl violet	Glia	↔
Di Rosa *et al.*^119^	NA	scz 11 ctr 13	scz 6/5 ctr 7/6	scz 66 ctr 68	scz 1	Fusiform gyrus	Cresyl violet	Glia	↔
Falkai and Bogerts^104^	VIBR	scz 13 ctr 11	scz 2/11 ctr 7/4	scz 43 ctr 43	scz 1	CA1#, 3*, 4,* PSB,* SB#	Nissl	Glia	↓*↔#
Falkai *et al.*^118^	VIBR	scz 13 ctr 11	scz 11/2 ctr 7/4	scz 43 ctr 43	NA	EC	Nissl	Glia	↔
Hoistad *et al.*^132^	NA	scz 13 ctr 13	scz 13/0 ctr 13/0	scz 52 ctr 52	scz 3	ACC (BA24)	Gallocyanin	Glia	↔
Jonsson *et al.*^128^	NA	scz 4 ctr 8	scz 4/0 ctr 8/0	scz 82 ctr 77	NA	HPC	Cresyl violet	Glia	↔
Kurumaji *et al.*^111^	NA	scz 13 ctr 10	scz 8/5 ctr 7/3	scz 60 ctr 67	NA	PFC#, TC#, OC*, PC*, PU*, CT#, SN#, PL#, TH#	Receptor binding assay	[^3^H] PK11195 binding (gliosis)	↓*↔#
Nasrallah *et al.*^135^	NIMH	escz 11 lscz 7 ctr 11	Na	escz 66 lscz 73 ctr 64	NA	CO	Hematoxylin-eosin stain	Glia	↔
Ongur *et al.*^117^	SFNC	scz 11 ctr 11	scz 7/3 ctr 7/4	scz 40 ctr 39	scz 4	sg24	Nissl	Glia	↔
Pennington *et al.*^126^	SFNC	scz 15 ctr 15	scz 9/6 ctr 9/6	scz 46 ctr 48	scz 4	Insular cortex	Cresyl violet	Glia	↔
Rajkowska *et al.*^115^	HTBRC, NIMH, UZ	scz 9 ctr 10	scz 7/2 ctr 6/4	scz 41 ctr 44	scz 5	PFC (BA9), OC (BA17)	Nissl	Glia	↔
Selemon *et al.*^113^	HTBRC, NIMH, UZ	scz 16 ctr 19	scz 12/4 ctr 10/9	scz 40 ctr 47	scz 10	PFC (BA9), OC (BA17)	Nissl	Glia	↔
Selemon *et al.*^116^	HTBRC, UZ	scz 9 ctr 10	scz 6/3 ctr 7/3	scz 44 ctr 48	scz 5	PFC (BA9,46)	Nissl	Glia	↔
Selemon *et al.*^114^	HBTRC, NIMH	scz 9 ctr 14	scz 6/3 ctr 10/4	scz 56 ctr 54	scz 3	FC (BA44) and DLPFC (BA9)	Nissl	Glia	↔
Selemon *et al.*^131^	SFNC	scz 15 ctr 15	scz 9/6 ctr 9/6	scz 45 ctr 48	scz 4	Lateral geniculate nucleus	Nissl	Glia	↔
Stark *et al.*^105^	NA	scz 12 ctr 14	scz 7/5 ctr 7/7	scz 70 ctr 69	scz 1	ACC (BA24)*, BA32#	Giemsa stain	Glia	↓*↔#
Stevens^102^	SEH	scz 28 ctr 18	scz 13/15 ctr 11/7	scz 41 ctr 37	NA	Multiple brain regions	Holzer's Technique	Glia	↑

Abbreviations: ACC, anterior cingulate cortex; AG, amygdala; BA, Brodmann area; CA, cornu ammonis; CO, corpus callosum; CT; caudate; ctr, control; DBC; Dusseldorf Brain Collection, DLFPC; dorsolateral prefrontal cortex; EC, entorhinal cortex; escz, early onset schizophrenia; FC, frontal cortex; HBTRC; Harvard Brain Tissue Resource Centre; HPC, hippocampus; lscz; late onset schizophrenia; md, mood disturbance; MTN, mediodorsal thalamic nucleus; NA, not available; NAS, nucleus accumbens; OC, occipital cortex; OFC, orbitofrontal cortex; PC, parietal cortex; PFC, prefrontal cortex; PL, pallidum; PSB, presubiculum; PU, putamen; SB, subiculum; scz, schizophrenia; SFNC; Stanley Foundation Neuropathology Consortium; sg, subgenual prefrontal cortex; SN; substantia nigra; ST, striatum; SEH, ST. Elizabeth's Hospital; TC, temporal cortex; TH, thalamus; UZ, University of Zagreb; VIBR, Vogt Institute of Brain Research; WBC, Würzburg Brain Collection.

*, # indicate which variables results are representing.

**Table 4:** Cytokine and chemokine in postmortem schizophrenia brain

*Author*	*Brain bank*	n	*Sex (m/f)*	*Age*	*Death from suicide*	*Brain region*	*Technique*	*Inflammatory markers*	*Results*
Dean *et al.*^138^	VBBN	scz 19 ctr 20	scz 15/4 ctr 16/4	scz 48 ctr 47	scz 8	DLPFC (BA46), ACC (BA24)	WB, PCR	sTNF-α#, tmTNF-α#, TNF-α receptor 1*,2#	↑*↔#
Durrenberger *et al.*^98^	BBPDGU	scz 10 ctr 10	scz 5/5 ctr 5/5	scz 66 ctr 61	NA	TL (BA22)	PCR	IL-13RA1	↓.
Fillman *et al.*^86^	NSWTRC	scz 37 ctr 37	scz 24/13 ctr 30/7	scz 51 ctr 51	NA	DLPFC (BA46)	PCR, WB, IHC	IL-8*, IL-6*, IL-1β#	↑*↔#
Fillman *et al.*^139^	SMRIAC	scz 35 ctr 35	scz 26/9 ctr 26/9	scz 43 ctr 44	scz 7	Middle frontal gyrus	PCR	IL-6#, IL-8*, IL-1β#, IL18#, TNF-α#	↓*↔#
Harris *et al.*^137^	SMRIAC	scz 35 ctr 33	scz 26/9 ctr 25/8	scz 43 ctr 45	NA	BA10	ELISA	IFN-γ	↑
Nakatani *et al.*^96^	VIFM	scz 7 ctr 7	scz 3/4 ctr 3/4	scz 61 ctr 61	scz 1	DLPFC (BA46), PC (BA40)	PCR	CCL3	↓
Rao *et al.*^73^	HBTRC	scz 10 ctr 10	scz 6/4 ctr 7/3	scz 59 ctr 49	NA	FC (BA10)	PCR, WB	TNF-α, IL-1β	↑
Schmitt *et al.*^94^	BBPDGU	scz 10 ctr 10	scz 5/5 ctr 8/2	scz 66 ctr 61	NA	TC (BA22)	PCR	IL-8*, IL-1α*, CCL2#, IL-1β#	↓*↔#
Toyooka *et al.*^136^	NA	scz 22 ctr 23	scz 16/6 ctr 14/9	scz 59 ctr 66	NA	PFC (BA46)*, posterior hypothalamic region, PC (BA 1-3), PU	PCR, WB	IL−1β #, IL-1RA*	↓*↔#
Volk *et al.*^155^	ACOME	scz 62 ctr 62	scz 47/15 ctr 47/15	scz 48 ctr 49	scz 16	PFC (BA9)	PCR	IL-1β*, IL-6*, IL-8#, IFN-β*	↑*↔#

Abbreviations: ACC, anterior cingulate cortex; BA, ACOME, Allegheny County Office of the Medical Examiner; Brodmann area; BBPDGU, Brain Bank for Psychiatric Diseases at the Gottingen University, CCL, chemokine (c-c motif) ligand; CCR, chemokine (c-c motif) receptor; ctr, control; DLFPC; dorsolateral prefrontal cortex; ELISA; enzyme-linked immunoadsorbent assay; FC, frontal cortex; HBTRC; Harvard Brain Tissue Resource Centre; IHC, IFN, interferon; IL, interleukin; NA, not available; NSWTRC; New South Wales Tissue Resource Centre; PC, parietal cortex; PFC, prefrontal cortex; PU, putamen; PCR, polymerase chain reaction; scz, schizophrenia; SMRIAC, Stanley Medical Research Institute Array Collection; sTNF, soluble TNF; TC, temporal cortex; TL, temporal lobe; tmTNF, transmembrane TNF; TNF, Tumor necrosis factor; VBBN, Victorian Brain Bank Network; VIFM, Victorian Institute of Forensic Medicine; WB, western blot.

*, # indicate which variables results are representing.

**Table 5:** Arachidonic acid cascade in postmortem schizophrenia brain

*Author*	*Brain bank*	n	*Sex (m/f)*	*Age*	*Death from suicide*	*Brain region*	*Technique*	*Inflammatory markers*	*Results*
Durrenberger *et al.*^98^	BBPDGU	scz 10 ctr 10	scz 5/5 ctr 5/5	scz 66 ctr 61	NA	Temporal lobe (BA22)	PCR	ALOX5AP	↓.
Fillman *et al.*^86^	NSWTRC	scz 37 ctr 37	scz 24/13 ctr 30/7	scz 51 ctr 51	NA	DLPFC (BA46)	PCR	PTGS2	↔
Fillman *et al.*^139^	SMRIAC	scz 35 ctr 35	scz 26/9 ctr 26/9	scz 43 ctr 44	scz 7	Middle frontal gyrus	PCR	PTGS2	↔
Maida *et al.*^140^	SFNC	scz 15 ctr 15	scz 9/6 ctr 9/6	scz 45 ctr 48	scz 4	PFC (BA8)*, TC (BA21 and BA22)# OC (BA18)#	WB, IHC	COX-1#, COX-2 #, cPGE_2_*	↓*↔#
Rao *et al.*^73^	HBTRC	scz 10 ctr 10	scz 6/4 ctr 7/3	scz 59 ctr 49	NA	FC (BA10)	PCR, WB	COX-1#, COX-2*, LOX5#, LOX12#, LOX15#, cPLA_2_*, sPLA_2_*, iPLA_2_#, cPGES#, mPGES#	↑*↔#
Tang *et al.*^141^	VBBN	scz 38 ctr 38	NA	scz 43 ctr 44	NA	DLPFC (BA46)	PCR	PTGS1, PTGS2, PTGER3, CYP4Z1	↔
Yokota *et al.*^142^	NA	scz 17 ctr 22	scz 12/5 ctr 13/9	scz 69 ctr 71	scz 0	HPC	IHC	COX-2	↔

Abbreviations: ALOX5AP, 5-lypoxygenase activating protein; BA, Brodmann Area; BBPDGU, Brain Bank for Psychiatric Diseases at the Gottingen University; ctr, control; COX, cyclooxygenase; cPGE, cytosolic prostaglandin E; FC, frontal cortex; CYP; cytochrome P450; HBTRC, Harvard Brain Tissue Resource Centre; IHC, immunohistochemistry; LOX, lipoxygenase; NA, not available; NSWTRC, New South Wales Tissue Resource Centre; OC, occipital cortex; PCR, polymerase chain reaction; PLA, phospholipase; PFC, prefrontal cortex; PGES, prostaglandin E synthase; PTGS, prostaglandin endoperoxide synthase; PTGER, prostaglandin E receptor 3; scz, schizophrenia; SFNC, Stanley Foundation Neuropathology Consortium; SMRIAC; Stanley Medical Research Institute Array Collection, TC, temporal cortex; VBBN, Victorian Brain Bank Network; WB, western blot.

*, # indicate which variables results are representing.

**Table 6:** Substance P in postmortem schizophrenia brain

*Author*	*Brain bank*	n	*Sex (m/f)*	*age*	*Death from suicide*	*Brain region*	*Technique*	*Inflammatory markers*	*Results*
Burnet and Harrison^151^	SFNC	scz 13 ctr 14	scz 8/5 ctr 9/5	scz 44 ctr 47	scz 3	ACC	AR	[^125^I]BH–substance P binding (NK1 receptor)	↔
Carletti *et al.*^143^	SFNC	scz 14 ctr 15	scz 9/5 ctr 9/6	scz 44 ctr 48	scz 4	AG*, TC#	*In situ* hybridization binding assay	Preprotachykinin A*, NK1 receptor#	↓*↔#
Harrington *et al.*^144^	NA	scz 4 ctr 5	scz 0/4 ctr 4/1	scz 71 ctr 69	NA	CT, PU	*In situ* hybridization	Preprotachykinin A	↔
Iadarola^145^	DCCO	scz 12 ctr 9	scz 10/2 ctr 8/1	scz 33 ctr 45	scz 9	SN	RIA	Substance P	↔
Kleinman *et al.*^146^	DCCO	scz 40 ctr 18	NA	scz 48 ctr 50	NA	FC, CT, NAS, PU, GB, HPL	RIA	Substance P	↔ (increased in other non schizophrenia psychotic disorders)
Rioux *et al.*^150^	NA	scz 5 ctr 5	NA	scz 70 ctr 70	NA	NAS#, PU*, CT#	AR	[^125^I]BH–substance P binding (NK1 receptor)	↔*↑#
Roberts *et al.*^149^	NA	scz 14 ctr 12	scz 8/6 ctr 7/5	scz 62 ctr 82	scz 2	TC (BA21/22)*, FC (BA4)*, PC (BA7)*, CI (BA24)*, HPC#, AG*, TH*, BG*	RIA	Substance P	↔*↑#
Tooney *et al.*^152^	NSWTRC	scz 6 ctr 6	scz 6/0 ctr 5/1	scz 44 ctr 43	scz 2 ctr 3	PFC (BA9)	IHC	NK1 receptor	↑ (except layer VI)
Toru *et al.*^148^	NA	scz 14 ctr 10	scz 9/5 ctr 7/3	scz 58 ctr 67	NA	BG*, SN*, TH*, HPC*, TC*, PFC*, PC*, OFC#	RIA	Substance P	↔*↑#
Weidenhofer *et al.*^153^	NSWTRC	scz 12 ctr 15	scz 10/2 ctr 13/2	scz 48 ctr 48	scz 3	AG	IHC, PCR	NK1 receptor	↔
Zech and Bogerts^147^	NA	scz 6 ctr 5	scz 1/5 ctr 3/2	scz 31 ctr 45	NA	BG	IHC	Substance P	↔

Abbreviations: ACC, anterior cingulate cortex; AG, amygdala; AR, autoradiography; BA, Brodmann Area; BG, basal ganglia; CI; cingulate cortex; CT, caudate; ctr, control; DCCO, District of Colombia Coroner's Office; FC, frontal cortex; HPC, hippocampus; HPL, hypothalamus; IHC, immunohistochemistry; NA, not available; NAS, nucleus accumbens; NK1, neurokinin 1; NSWTRC, New south wales tissue resource centre; OFC, orbitofrontal cortex; PC, parietal cortex; PCR, polymerase chain reaction; PFC, prefrontal cortex; PU, putamen; RIA, radioimmunoassay; scz, schizophrenia; SN, substantia nigra: SFNC, Stanley Foundation Neuropathology Consortium; TC, temporal cortex; TH, thalamus.

*, # indicate which variables results are representing.

**Table 7:** Other markers in postmortem schizophrenia brain

*Author*	*Brain bank*	n	*Sex (m/f)*	*Age*	*Death from suicide*	*Brain region*	*Technique*	*Inflammatory markers*	*Results*
Arion *et al.*^157^	UPCNMDBB	scz 14 ctr 14	scz 12/2 ctr 12/2	scz 43 ctr 42	scz 3	PFC (BA9)	PCR	SERPINA3, IFITM1, IFITM3, CHI3L1, HSPB1, MT2A	↑
Catts and Weickert^161^	SMRIAC, NSWTRC	scz 72 ctr 71	scz 50/22 ctr 55/16	scz 47 ctr 48	scz 15	DLPFC*, OFC#	PCR	TNFSF13	↑*, ↔#
Durrenberger *et al.*^98^	BBPDGU	scz 10 ctr 10	scz 5/5 ctr 5/5	scz 66 ctr 61	NA	TL (BA22)	PCR	TIMP1, TNFRSF1A, TYROBP	↓
Fillman *et al.*^86^	NSWTRC	scz 37 ctr 37	scz 24/13 ctr 26/9	scz 51 ctr 48	NA	DLPFC (BA46)	PCR	NFκB#, SERPINA3*, IL6ST#	↑*↔#
Fillman *et al.*^139^	SMRIAC	scz 35 ctr 35	scz 26/9 ctr 26/9	scz 43 ctr 44	scz 7	Middle frontal gyrus	PCR	SERPINA3*, IL-1 RL1#	↑*↔#
Harris *et al.*^137^	SMRIAC	scz 35 ctr 33	scz 26/9 ctr 25/8	scz 43 ctr 45	NA	BA10	ELISA	TIMP1	↔
Hwang *et al.*^77^	SNFC, SMRIAC	scz 33 ctr 34	scz 23/10 ctr 23/11	scz 44 ctr 46	NA	HPC	PCR	CD163, S100a8, S100a9, IFITM1, IFITM2, IFITM3	↑
Iwamoto *et al.*^158^	SFNC	scz 13 ctr 15	scz 8/5 ctr 9/6	scz 44 ctr 48	scz 4	PFC (BA10)	PCR	IFITM3	↑
Rao *et al.*^73^	HBTRC	scz 10 ctr 10	scz 6/4 ctr 7/3	scz 59 ctr 49	NA	FC (BA10)	PCR, WB	IL-1R#, NFκBp50*, NFκBp65*, iNOS#	↑*↔#
Saetre *et al.*^93^	SFNC, HBTRC, MBB	scz 55 ctr 55	NA	scz 59 ctr 55	NA	FC (BA8 and 9), superior frontal gyrus	PCR	IFITM2, IFITM3, SERPINA3, GPB1	↑
Schmitt *et al.*^94^	BBPDGU	scz 10 ctr 10	scz 5/5 ctr 8/2	scz 66 ctr 61	NA	TC (BA22)	PCR	LPL*, CFD*, PTGER4*, EDG3* ITGA1#, LCP1#, LTC4S#, MTHFD2#, SOD2#, CCR1#, IL1RAP#, IFI16#, IFNAR2#, CD84#, GPX#	↓* ↔#
Shao and Vawter^160^	SMRIAC	scz 32 ctr 27	scz 23/9 ctr 23/6	scz 43 ctr 44	NA	DLPFC (BA46)	PCR	TNFSF8, TNFSF10	↔
Siegel *et al.*^159^	ACOME	scz 57 ctr 57	scz 42/15 ctr 42/15	scz 47 ctr 48	scz 16	PFC (BA9)	PCR, *in situ* hybridization	IFITM1, IFITM2/3	↑
Sun *et al.*^156^	SFNC	NA	NA	NA	NA	FC	PCR	NFκB2	↔
Thomas *et al.*^154^	SFNC	scz 15 ctr 15	scz 9/6 ctr 9/6	scz 45 ctr 48	scz 4	DLFPC (BA9,46), ACC (BA24)	IHC	ICAM-1	↔
Volk *et al.*^155^	ACOME	scz 62 ctr 62	scz 47/15 ctr 47/15	scz 48 ctr 49	scz 16	PFC (BA9)	PCR	NFκB1#, NFκB2#, Shn-2*	↓*↑#

Abbreviations: ACC, anterior cingulate cortex; ACOME, Allegheny County Office of the Medical Examiner; APOL, apoliprotein L; BA, Brodmann area; BBPDGU, Brain Bank for Psychiatric Diseases at the Gottingen University; CCR, chemokine (c-c motif) receptor: CD, cluster of differentiation; CFD, complement factor D; CHI3L1, chitinase-3 like protein 1; ctr, control; DLFPC; dorsolateral prefrontal cortex; EDG3, endothelial differentiation, sphingolipid g-coupled-receptor; FC, frontal cortex; GPX, glutathione peroxidase; GPB, guanylate binding protein; HBTRC, Harvard Brain Tissue Resource Centre; HPC, hippocampus; HSPB, heat shock protein beta; ICAM, intercellular adhesion molecule; IFI, interferon gamma-inducible; IFITM; interferon-induced transmembrane; IFNAR, interferon (alpha, beta and omega) receptor: IHC, immunohistochemistry; IL1RAP; interleukin 1 receptor accessory protein; IL1RL, interleukin 1 receptor like; IL6ST, glycoprotein 130; ITGA, integrin alpha; iNOS, inducible nitric oxide; LCP, lymphocyte cytosolic protein; LPL, lipoprotein lipase; LTC4S, leukotriene C4 synthase; MBB, Maudsley Brain Bank; MT2A, metallothionein 2A; MTHFD, methylenetetrahydrofolate dehydrogenase; NA, not available; NFκB, nuclear factor kappa-light-chain-enhancer of activated B cells; NSWTRC; New South Wales Tissue Resource Centre; OFC, orbitofrontal cortex; PCR, polymerase chain reaction; scz, schizophrenia; PFC, prefrontal cortex; PTGER, prostaglandin E receptor 4; SERPIN, serine protease inhibitor; SFNC; Stanley Foundation Neuropathology Consortium; Shn-2, Schnurri-2; SMRIAC, Stanley Medical Research Institute Array Collection; SOD, superoxide dismutase; TC, temporal cortex; TIMP; tissue inhibitor of metalloproteinases; TL, Temporal lobe; TNFSF, tumor necrosis factor superfamily; TYROBP, TYRO protein tyrosine kinase binding protein; UPCNMDBB, University of Pittsburgh's center for the neuroscience of mental disorder brain bank.

*, # indicate which variables results are representing.
